# The relationship of phonological ability, speech perception, and auditory perception in adults with dyslexia

**DOI:** 10.3389/fnhum.2014.00482

**Published:** 2014-07-02

**Authors:** Jeremy M. Law, Maaike Vandermosten, Pol Ghesquiere, Jan Wouters

**Affiliations:** ^1^Faculty of Psychology and Educational Sciences, Parenting and Special Education Research Unit, KU LeuvenLeuven, Belgium; ^2^Laboratory for Experimental ORL, Department of Neuroscience, KU LeuvenLeuven, Belgium

**Keywords:** dyslexia, literacy, phonological processing, speech perception, auditory processing, amplitude rise time, frequency modulation

## Abstract

This study investigated whether auditory, speech perception, and phonological skills are tightly interrelated or independently contributing to reading. We assessed each of these three skills in 36 adults with a past diagnosis of dyslexia and 54 matched normal reading adults. Phonological skills were tested by the typical threefold tasks, i.e., rapid automatic naming, verbal short-term memory and phonological awareness. Dynamic auditory processing skills were assessed by means of a frequency modulation (FM) and an amplitude rise time (RT); an intensity discrimination task (ID) was included as a non-dynamic control task. Speech perception was assessed by means of sentences and words-in-noise tasks. Group analyses revealed significant group differences in auditory tasks (i.e., RT and ID) and in phonological processing measures, yet no differences were found for speech perception. In addition, performance on RT discrimination correlated with reading but this relation was mediated by phonological processing and not by speech-in-noise. Finally, inspection of the individual scores revealed that the dyslexic readers showed an increased proportion of deviant subjects on the slow-dynamic auditory and phonological tasks, yet each individual dyslexic reader does not display a clear pattern of deficiencies across the processing skills. Although our results support phonological and slow-rate dynamic auditory deficits which relate to literacy, they suggest that at the individual level, problems in reading and writing cannot be explained by the cascading auditory theory. Instead, dyslexic adults seem to vary considerably in the extent to which each of the auditory and phonological factors are expressed and interact with environmental and higher-order cognitive influences.

## Introduction

Dyslexia is a neurological condition affecting 5–10% of the population. This specific learning disability impacts an individual's ability in learning to read and write despite adequate intelligence, education, and remediation (Vellutino et al., 2004). It has been well established in the literature that the major causes of the expressed literacy problems lay within a deficit in the phonological domain, specifically in the quality and accuracy of phonological representations (Snowling, 2000). In this paper the auditory temporal processing deficit theory of dyslexia, and its cascading effects on speech and phonological processing will be examined. To this end, measures of slow-rate modulation, and speech perception will be assessed along with phonological and literacy measures in a population of university level dyslexic and non-dyslexic adult readers.

A vital part in the development of phonological representations is the awareness of how speech sounds correspond to a written symbol. Findings of the past few decades have begun to suggest the existence of an underlying deficit in low-level auditory temporal processing within the dyslexic population (Farmer and Klein, 1995; Habib, 2000; Boets et al., 2006). Thus, if dyslexic readers perceive speech or related auditory cues inaccurately, the mapping of speech sounds onto their corresponding symbols will be problematic.

Beginning with Tallal's (1980) study of temporal order judgment of children with specific language impairments, research has explored the idea that the primary deficit of dyslexics could lay in deviant auditory processing skills. Early research related the interpretation of “temporal processing” restrictively to rapid succession or short durational cues (e.g., Tallal, 1980). However, recent studies have demonstrated that the deficits observed in dyslexic readers are not merely limited to the processing of short, rapidly presented stimuli, but also to slow-rate dynamic acoustic stimuli such as frequency modulations (FMs) and sound rise time discrimination (RT). Such a deficit has been theorized to produce a cascade ultimately disrupting an individual's reading and spelling abilities. If an individual were to be affected by poor auditory processing of slow-rate modulations (between 2 and 20 Hz), it would be expected that speech perception would ultimately be affected, since the identification of phonemes and syllables depends on changes in the amplitude that occur respectively around 50 ms (i.e., 20 Hz) to 500 ms (i.e., 2 Hz). Such speech perception difficulties could impact the segmentation of aspects of the speech signal into smaller elements, thus hampering the development of phonological representations and ultimately disrupting the creation of accurate mapping schemes between speech sound and corresponding graphemes (Poelmans et al., 2011). Ultimately, these poor phoneme-grapheme representations will be expressed as poor coding and decoding abilities impacting word reading and spelling.

Slow-rate auditory modulations can be assessed by two different tasks, FM and rise time (RT) detection task. FM detection assesses the individual's ability to detect frequency fluctuations in a carrier frequency at a certain modulation rate. Such FMs could be said to represent the fine structure found within the envelopes of the speech waveform (Rosen, 1992). Research on FM detection of dyslexics and controls have found significant group differences, where dyslexics have been shown to have a reduced sensitivity compared to controls, thus demonstrating FM task's ability to differentiate between adult, school aged, and pre-reading dyslexics from normal readers (Witton et al., 1998, 2002; Ramus et al., 2003; Boets et al., 2007). Yet, of the 12 papers examining FM perception in a review study by Hämäläinen et al. (2013), three of the studies were not able to replicate these group differences (Halliday and Bishop, 2006; Stoodley et al., 2006; White et al., 2006).

In addition to findings of group differences, a study by Witton et al. (1998) found phonological decoding skills of both dyslexics and controls to be significantly correlated with FM sensitivity of 2 and 40 Hz. The review paper by Hämäläinen et al. (2013) noted 8 separate studies that reported correlations between FM detection thresholds and reading and/or spelling skills. Yet, 3 studies were unable to replicate these results (Van Ingelghem et al., 2005; Heath et al., 2006; Dawes et al., 2009).

An alternative measure of auditory processing that taps into aspects of slow-rate dynamic processing mechanisms and that has been indicated to be a sensitive measure in discriminating between populations of dyslexic and normal readers is rise time discrimination (RT). Rise time, in comparison with FM tasks, measures the larger grain size of the speech waveform, which focuses specifically on the speech envelope (Rosen, 1992). Specifically, the RT task accesses an individual's ability to detect subtle differences in the rate of change of an amplitude envelope. The perceptions of such cues are utilized in the segmentation of the speech signal into its base parts, such as syllables or onsets and rhymes, which is necessary for speech perception (Goswami et al., 2010). Detection of such cues has been shown to be significantly associated with reading, writing and phonological skills in an adult population (Hämäläinen et al., 2005). Goswami et al. (2002) demonstrated that 25% of unique variance in reading and spelling in children could be predicted by individual differences in rise time sensitivity, with IQ and age being controlled for. Findings demonstrating RT's relation to reading have also remained consistent across different orthographies (Goswami et al., 2011). When comparing persons with dyslexia to typical readers, child studies have demonstrated consistent group differences in RT perception across various measurement techniques (for a review see Hämäläinen et al., 2013; note the exception of Hämäläinen et al., 2009). On the other hand, adult studies have not been so clear. Despite some adult studies showing significant poorer performance on RT tasks in adults with dyslexia (Hämäläinen et al., 2005; Thomson et al., 2006; Corriveau et al., 2007), findings vary between the different measurement techniques employed (see Thomson et al., 2006; Pasquini et al., 2007). Traditionally, pure tone carrier signals are modulated in RT-tasks, but this lacks important frequencies of real speech. Hence, they do not activate a broader frequency region in the auditory system compared to speech weighted noise signals. In an effort to mimic the demand of real speech within the RT detection measure, Poelmans et al. (2011) utilized a single ramp rise time discrimination task that consists of a speech-weighted noise with a linear amplitude rise time. They showed that the application of a speech weighted noise signal resulted in reliable performance in children and did not produce any ceiling or floor effects, which differed from pilot studies of pure tone carrier signals.

However, not all auditory processing aspects seem to be impaired in dyslexic readers. In contrast to slow-rate dynamic auditory processing (RT, FM), intensity discrimination (ID) does not display group differences between typical and dyslexic readers (for a review see Hämäläinen et al., 2013). This suggests that related task demands, attention and cognitive aspects are not the driving factor of the observed auditory problems since they are equal across RT, FM, and ID tasks. In addition, as the RT measure includes changes of intensity over time, the lack of group differences on the ID tasks suggests that a poorer performance on the RT-task is not a reflection of difficulties in ID ability but rather of the changes in intensity.

An understanding of slow-rate dynamic modulations such as RT and FM is important due to their prevalence in the speech signal, appearing at various grain sizes of phonological information ranging from intonation, onset and rhyme to the phoneme. If an individual has a deficit in processing these modulations, it is believed that it would be expressed in their ability to perceive speech.

Most often speech sound processing of dyslexics is assessed through the use of a categorical perception measure. Studies utilizing categorical perception tasks have demonstrated that subjects with dyslexia possess a reduced capacity for perception and categorization of phonemes (for a review see Vandermosten et al., 2010, 2011). However, results from such tasks are often restricted to a subset of the dyslexic population sampled (Manis et al., 1997; Adlard and Hazan, 1998) or to a specific speech condition or task (Maassen et al., 2001; Blomert and Mitterer, 2004). Typically, categorical perception tasks utilize optimal listening conditions. Such conditions allow for compensation of specific deficits in phoneme identification (Manis et al., 1997; Assmann and Summerfield, 2004; Ziegler et al., 2009). Although speech-in-noise tasks are influenced by higher-order cognitive processes such as lexical and phonotactic knowledge, they provide a more ecological and natural measure of speech sound processing than categorical perception. By presenting speech stimuli in the presence of a masking noise, a participant's ability to identify and comprehend real speech sounds under varying noise-masking scenarios is assessed. The ability to identify speech-in-noise requires the individual to separate out the background noise from the target speech signal. This isolation allows for the individual to produce precise representations of the rapidly evolving spectral information. It has been shown that, although all listeners demonstrate some reduced capacity for perception under noisy background conditions, dyslexic children (Snowling et al., 1986; Wible et al., 2002; Bradlow et al., 2003; Ziegler et al., 2005, 2009; Boets et al., 2011) and dyslexic adults (Dole et al., 2012) exhibit pronounced difficulty with this task while often not demonstrating any impairment of speech perception in silent conditions (Brady et al., 1983; Bradlow et al., 2003). Yet, Hazan et al. (2009) were not able to replicate these findings in an adult population.

Although studies have demonstrated deficits independently in the slow-rate dynamic processing and speech-in-noise perception in individuals with dyslexia, only two studies have assessed both of these measures of signal processing in the same population (Boets et al., 2011; Poelmans et al., 2011). Boets et al. retrospectively explored this relationship in a population of preschool children who later developed dyslexia and showed that these children were already impaired in slow-rate FM sensitivity and speech perception prior to reading instruction. These pre-reading measures were also found to relate to each other and uniquely predicted later growth in reading. A more recent study by Poelmans et al. (2011), which followed up the same students of Boets, in 6th-grade children showed no clear evidence supporting relations between slow-rate dynamic auditory processing and speech perception itself. Given that this correlation was present at an earlier age (Boets et al., 2011), this might suggest that the link between auditory and speech perception skills is disappearing through development. However, more validation in adult participants is needed.

Although studies such as that of Boets and colleagues have found support for the auditory temporal processing deficit theory of dyslexia, the theory is not without its controversy. Criticism has arisen from the heterogeneity of the found deficits. It has been suggested that differences between group means are a reflection of a small number of poor performing dyslexic subjects. Ramus et al. (2003) examined an adult population and noted that auditory deficits were limited to only 39% of the subjects with dyslexia and that auditory processing had only a weak correlation with phonology and reading. Other criticisms have suggested that general difficulties with task completion might underlie the poor performance of subjects with dyslexia in psychophysical studies and lead researchers to misinterpret non-sensory difficulties as sensory ones (Stuart et al., 2001; Roach et al., 2004).

Our study will investigate the different levels of processing skills (i.e., auditory, speech-in-noise perception, and phonological processing) in one and the same sample of dyslexic and normal reading adults. So far, such an integrative approach has not been applied to adults, despite being vital to understand the interrelations between auditory processing, speech perception, phonological processing, and reading (problems). Furthermore, in contrast to previous studies, our study will not only investigate the interrelation between these skills and compare performance between groups, but we will also examine the individual level deviance scores.

Given that dyslexia is a disability measured and defined as deviant performance, research should reflect this by demonstrating a substantial number of individuals whose performance significantly differs from normal performance (Ramus et al., 2003; Heath et al., 2006; Ziegler et al., 2008; Hazan et al., 2009). As noted in Hazan et al. (2009) group comparisons could potentially mask significant individual differences or highlight differences which may not essentially be deviant, hence it is not sufficient in dyslexia research to merely demonstrate significant group differences without investigating the individual deviance scores. In addition, according to the auditory deficit theory, dyslexic readers should show consistent deficiencies across each level of processing, otherwise phonological impairments are presumably not secondary to speech and lower-level auditory problems.

Given that performance in adults is more prone to compensational mechanisms, the slow-rate dynamic tasks (FM and RT) will be assessed together with a control measure for attention and task complexity (ID). Although the inclusion of such well-matched control task helps in distinguishing effects of task demands from true effects, so far no study has included them as a control within all levels of statistical analyses. A few studies have included a control variable for attention and task related demands in group matching (Hämäläinen et al., 2005; Thomson et al., 2006; Pasquini et al., 2007), yet this does not prevent individual variation in groups exhibiting a significant role in relationships between psychophysical, phonological, and literacy measures.

In sum, this study will address three main questions: (i) Do adults with dyslexia demonstrate deficits in auditory processing, speech perception, and phonological abilities at the group level and at the individual level? (ii) Does a close relationship exist between the auditory processing, speech perception, and phonological skills or do they rather contribute independently to reading skills? (iii) Based on individual deviance analyses, do the same participants display deviant scores across the three skills (i.e., auditory processing, speech perception, and phonological processing)?

To achieve this, auditory processing skills will be assessed by two slow-rate modulation tasks, i.e., RT and FM, and by a control task, i.e., ID. Speech perception will be assessed by a word and sentences in noise task. Lastly, phonological processing will be accessed through the classical threefold of phonological awareness (PA), verbal short-term memory (VSTM), and rapid automatic naming (RAN) tasks.

## Materials and methods

### Participants

A total number of 90 undergraduate students were recruited for this study, 54 (36 female and 18 male) non-dyslexic and 36 (26 female and 10 male) participants with dyslexia. In order to participate, the dyslexic students needed to have a diagnosis completed by a registered and qualified clinical psychologist in secondary school or earlier and had to be registered at the office of Student Development & Services. The fact that the adults with dyslexia were selected from a university population, a higher level of reading achievement is expected than in a general sample of individuals of the same age, due to the selectivity of universities. This is reflected in some dyslexic student's normal reading and spelling scores as seen in Table 1. Based on their higher than expected literacy scores these participants may be considered as “compensated” dyslexics. Research has shown that strengths in cognitive abilities, such as the use of contextual cues (Frith and Snowling, 1983; Nation and Snowling, 1998), semantic knowledge (Snowling et al., 2000), visual memory (Campbell and Butterworth, 1985), and morphological knowledge (Elbro and Arnbak, 1996) help this group of individuals with dyslexia to minimize the expression of their reading difficulties.

**Table 1:** **Participant characteristics**.

**Measure**	**NR**		**DYS**		***t***	***p***
	***M***	***SD***	***M***	***SD***		
Age (years)	22.0	3.0	21.8	4.8	0.227	1
Non-Verbal IQ (APM)	112.7	9.9	107.0	20.7	1.777	0.158
**LITERACY**						
Word-reading^a^ (SS) (WRAT-III)	106.1	5.8	91.7	10.1	8.575	<0.002
Spelling^a^ (SS) (WRAT-III)	107.6	6.6	90.8	8.8	10.305	<0.002
Literacy (*z*-score)	−0.1	1.1	−3.3	1.7	11.396	<0.001

*All p-values are Bonferroni adjusted for multiple comparisons. APM, Raven advanced progressive matrices; WRAT-III, Wide Range Achievement Test III*.

a *Scores are standardized (M = 100, SD = 15)*.

The non-dyslexic population were comprised of students who have no documentation or history of reading difficulty and whose word reading scores did not fall in the bottom 5% of the WRAT norms (Wilkinson, 1993). Recruitment of the dyslexic population for the study was made through the University's Student Services, while the control population was gathered based on class announcements and posters placed throughout each campus.

All participants were at least 18 years of age and attended one of three universities in Ontario, Canada. All participants were native English speakers without a history of brain damage, language problems, psychiatric symptoms or visual problems which could not be corrected for by a corrective lens. Additionally all participants had adequate audiometric pure-tone hearing thresholds for the test ear (i.e., 25 dB HL or less on 0.25–8.0 kHz) and adequate non-verbal IQ defined by a standard score greater than 85 on Raven's advanced progressive matrices. Table 1 shows participant characteristics for the two groups. Groups did not differ in age, gender, and non-verbal IQ.

### Tasks

#### Literacy

Literacy was assessed by the WRAT-III reading and spelling subtests (Wilkinson, 1993). The reading subtest required the subject to read aloud a list of 42 words. The subject received a single point for each correctly pronounced word to a maximum score of 42. The spelling subtest required the subject to accurately spell a series of dictated words. The words were presented orally by the test administrator preceding and following a sentence containing the target word. The test was scored by giving one point for each correctly spelled word to a maximum score of 40 points.

#### Phonological skills

Each domain of one's phonological skills, as represented in Wagner and Torgesen (1987), was individually tested.

*Phonological awareness* (PA) was assessed through the use of the Spoonerism subtest from the Phonological Assessment Battery (PhAB) (Frederickson et al., 1997). Spoonerism tasks have been demonstrated to be able to significantly differentiate between an adult dyslexic population and control groups (Ramus et al., 2003). This test of PA targeted onset-rhyme awareness and requires phoneme manipulation and deletion. This task involved two parts. The first required the participant to replace the first sound of a word with a new sound (e.g., cot with a /g/ gives “got”). In part two, word pairs were orally presented to the participant; in turn they were requested to transpose the onset of the sounds of the two words. For example, “plane crash” will become “crane plash” or “King John” becomes “Jing Kon.” Rate scores, measured in number of correct items per second, were calculated as the total correct responses divided by the total time to complete the task. Due to ceiling level being reached within the control group accuracy was not separately evaluated.

*Verbal short-term memory* was assessed by The Number Repetition (digit span forward) subtest from The Clinical Evaluation of Language Fundamentals 4th edition (CELF-4) (Semel et al., 2003). Digit span forward required the immediate serial recall of an orally presented series of digits. List length was incrementally increased from two to nine digits and presented orally at a rate of one digit per second. The test score was calculated as the total number of correctly recalled lists with a maximum score of 16.

Verbal short-term memory was also assessed by the non-word recall subtest from the Working Memory Test Battery (WMTB) (Pickering and Gathercole, 2001). For this task sequences of single syllable non-sense words were presented orally to the participants. Each participant was requested to repeat the sequence in the correct order. The list length was incrementally increased, from one to six words in length. Six trials were available for presentation at each list length. The task was discontinued when three errors were made in a given list length. The test score was calculated as the total number of correctly recalled lists with a maximum score of 36.

*Rapid Automatic Naming* (RAN) was assessed through two naming tasks. A color-naming test adapted from Boets et al. (2006) was selected. Five colors (black, yellow, red, green, and blue) were presented in 5 rows containing 10 color stimuli each. In addition, the object-naming subtest from The Phonological Assessment Battery (PhAB) (Frederickson et al., 1997) was used. Five line drawings of common objects (desk, ball, door, hat, box) were presented in 5 rows each containing 10 items. For both tasks participants were instructed to name aloud each of the objects or colors as quickly and as accurately as possible. A score of the number of symbols named per second was calculated.

#### Auditory processing and speech perception experimental setup

All tasks were conducted on campus and were administered individually in a private room, with minimal background noise and distraction. All auditory and speech perception tasks were performed on a Dell Latitude D510 and controlled by APEX software (Laneau et al., 2005; Francart et al., 2008). Speech perception and auditory processing stimuli were presented through Sennheiser HDA 200 headphones to the right ear. Auditory processing procedure and tasks were adapted from those used and described by Poelmans et al. (2011).

#### Auditory processing tasks

All auditory processing task thresholds were estimated by means of a one-up, two-down adaptive staircase procedure which is designed to target a threshold corresponding to 70.7% correct responses (Levitt, 1971). Tasks were presented within a three-alternative forced-choice, “odd-one-out” paradigm. In each trial three stimuli were presented requiring the participant to determine which sound differed from the others. An inter-stimulus interval of 350 ms was used. All tasks were terminated after ten reversals. Thresholds were the arithmetic mean of the last 4 reversals. Each participant completed two threshold runs of each task.

*FM-detection task* required participants to detect a 2 Hz sinusoidal FM of a 1 kHz carrier tone with varying modulation depth. The reference stimulus was a pure tone of 1 kHz. Modulation depth decreased by a factor of 1.2 from 100 to 11 Hz. At this point modulation depth decreases by a step size of 1 Hz. The length of both the reference and the target stimulus was 1000 ms including 50 ms cosine-gated onset and offset. The detection threshold was defined as the minimum depth of frequency deviation (in Hz) required to detect the modulation.

*Sound rise time discrimination* sensitivity consisted of a speech weighted noise with linear amplitude rise times. Rise times varied logarithmically between 15 and 500 ms in 41 steps. The total duration of the stimulus was fixed to 800 ms, including a linear fall time of 75 ms. The stimulus of 15 ms rise time was used as the reference stimulus for each trial. Discrimination thresholds were defined as the minimal difference in the rise time required discriminating between the reference and target stimulus.

*Intensity discrimination* task was identical to the FM and RT discrimination task in its presentation and procedure. Stimuli, of an 800 ms duration, consisting of a speech-weighted noise and a linear rise time and fall time of 75 ms were used. The stimulus of 70 dB SPL was utilized as a reference stimulus for each trial. Intensity was varied linearly between 70 and 80 dB SPL in 40 steps of 0.25 dB SPL each. Discrimination thresholds were defined as the minimal intensity difference (in dB SPL) required to discriminate between the reference and the target stimulus.

#### Speech-in-noise perception

Speech-in-noise intelligibility was assessed for both words and sentences. During testing, the speech level was varied while the background noise level was fixed at 70 dB SPL. To assess the association of RT and FM discrimination in speech perception, two speech-in-noise tasks were administered. The first dealing with words-in-noise which would require less reliance on rise time processing and more on FM and the second which included sentences in noise which would rely more heavily on RT discrimination to accurately decompose and segment the sentence into finer grained elements for processing.

*Words-in-noise* perception was assessed with The Computer Aided Speech Perception Assessment (CASPA) developed by Boothroyd (2006) (for application see McCreery et al., 2010). A random selection of 3 lists of 10 CVC words were presented orally by a female speaker against a competing speech weighted noise at varying signal-to-noise ratios (SNR) (−5, −10, and −13 dB). Each list contained a single occurrence of the same set of 30 phonemes (20 consonants and 10 vowels). A practice list of 0 dB SNR was first administered to the participant. Participants were instructed to repeat each target word after presentation; if the participant was unable to repeat the target word correctly they were instructed to repeat every perceived phoneme. The percentage of correctly perceived phonemes was calculated for each SNR. The Speech Reception Threshold (SRT) was calculated for each participant through fitting to the data a logistic function relating the percentage of correct responses to SNR level (for a similar approach see Poelmans et al., 2011).

Speech-in-noise intelligibility of sentences was assessed using stimuli adapted from The Hearing in Noise Test (HINT) (Nilsson et al., 1994). Speech material consisted of English sentences spoken by a male speaker. The HINT stimuli consisted of a 70 dB long-term average speech spectrum masking noise and 12 equivalent 20-sentence lists. Two lists were administered after one practice list was presented. Lists were randomly selected from the 12 available. In the HINT adaptive procedure, beginning at 58 dB, the presentation level of all sentences were adjusted by 2 dB steps. Speech-in-noise intelligibility thresholds for each participant were calculated by averaging the last 6 SNR. Final values for each measure were inverted by multiplying by a factor of −1 to obtain a positive correlation matrix and for the creation of *z*-sores.

### Statistical analyses

All data were checked with Shapiro-Wilk's test for normality. The assumption of homogeneity of variance was assessed by Levene's Test for Equality of Variances.

#### Individual deviance analyses of composite scores

A two-step process, as in Ramus et al. (2003) (also see Boets et al., 2006, 2007; Reid et al., 2007; Hazan et al., 2009), was used to create *z*-scores for each variable and to examine group differences in the proportion of deviant subjects on literacy tasks, phonological tasks, speech-in-noise perception, and dynamic auditory perception. As done in Ramus et al. (2003) a control mean and standard deviation were calculated for each measured variable based on the scores of the normal reading sample. However, any subject of the NR sample scoring below the set threshold of −1.65 *SD* (bottom 5% of the population) was removed to compute the final control mean and *SD*. This extra step was a means to prevent any inattentive or distracted control from exaggerating the normal range of performance. *Z*-scores for all subjects were then recalculated based on this new final control mean and *SD*. Individual deviance was calculated from these *z*-scores and defined as any subject falling below the −1.65 *SD* threshold. For the purposes of this paper the term deviancy score is referring only to those scores falling below this threshold. We do not imply any answer to the delay/deficit discussion concerning dyslexia. In acknowledgment of the possible exaggeration of the dyslexics' deficits by such a two-step method, the more strict threshold of −1.65 *SD* was chosen.

The resulting *Z*-scores were used to create composite scores. For each participant a literacy score was calculated by averaging the *z*-scores of the WRAT reading and spelling subtests (Literacy); a phonological awareness (PA) score was calculated as the *z*-score of the Spoonerism task, The two RAN *z*-scores were averaged into one overall RAN score (RAN). Digit span and non-word recall tasks were averaged to create a verbal short-term memory score (VSTM). Due to the lack of strength in the correlations found within the auditory processing and within speech perception measures no composite scores were created for these groups of variables.

#### Multiple comparison corrections

In order to avoid the possibility of making a false positive conclusion in group comparisons all reported *p*-values for *t*-tests and ANOVAs were adjusted using a Bonferroni correction, which entailed the multiplication of the given *p*-value by the total number of comparisons per question to a maximum Bonferroni adjusted *p*-value of 1. If the adjusted *p*-value remains less than the original alpha of 0.05 then the null hypothesis was rejected.

## Results

### Performance of dyslexic vs. normal reading adults

#### Literacy

Literacy results are presented in Table 1. There was a statistically significant difference in the mean scores of reading and spelling between groups, with the dyslexic group preforming significantly poorer, *t*_(50.283)_ = 8.575; *p* < 0.005, and *t*_(60.675)_ = 10.305; *p* < 0.005.

#### Phonological skills

Each domain of one's phonological skills, as represented in Wagner and Torgesen (1987), was tested. Phonological awareness (PA) was tested by the spoonerism task of the PhAB, verbal short-term memory (VSTM) by digit span and non-word recall and RAN by object and color naming. Test scores are presented in Table 2.

**Table 2:** **Phonological abilities: descriptive statistics and *t*- and *p*-values from independent *t*-tests**.

**Measure**	**NR**		**DYS**		***t***	***p***
	***M***	***SD***	***M***	***SD***		
Spoonerism (correct/s)	0.23	0.08	0.10	0.04	9.042	<0.005
Digit span	12.32	1.87	10.78	2.00	3.712	<0.005
Non-word recall	20.09	2.25	17.61	2.62	4.795	<0.005
RAN (color)	2.01	0.33	1.72	0.31	4.262	<0.005
RAN (object)	1.77	0.24	1.50	0.25	5.059	<0.005

*All p-values are Bonferroni adjusted for multiple comparisons*.

Independent sample *t*-tests were run to determine differences between groups in measures on phonological skills. Scores of the non-word recall and Spoonerism tasks were not found to be normally distributed. In order to approach a normal distribution they were transformed by a square root transformation. Adults with dyslexia were found to perform significantly poorer then controls on all measures.

#### Speech perception and auditory processing

In order to approach a normal distribution for more variables, the best score on the FM measure was transformed by a logarithmic transformation after the scores had been reversed, while the best score on the ID measure was transformed by the use of a square root transformation after the scores had been reversed, and the RT scores were transformed using a square root transformation (Field, 2009).

Since the aim of this research is to evaluate threshold estimations as an indicator of a subject's sensory capability, the two threshold trials were not averaged and instead the best score of each test was selected (for a similar approach see Boets et al., 2006). Threshold means and standard deviations of all auditory measures for each group can be found in Table 3.

**Table 3:** **Auditory and speech-in-noise measures: descriptive statistics and *t* and *p*-values from independent *t*-tests**.

**Measure**	**NR**		***DYS***		***t***	***p***
	***M***	***SD***	***M***	***SD***		
FM (Hz)	3.82	1.38	4.58	2.38	−1.922	0.174
RT (ms)	73.07	47.41	117.22	65.94	−3.695	0.003
ID (dB)	1.04	0.54	1.46	0.76	−3.100	0.009
HINT (SRT in dB)	−3.03	0.93	−3.11	0.91	−0.373	1
CASPA (SRT in dB)	−11.06	0.92	−11.01	1.02	0.243	1

*All p-values are Bonferroni adjusted for multiple comparisons*.

Results demonstrated that dyslexic readers scored significantly poorer on measures of RT discrimination and ID, but not on FM-detection nor on the two tasks for speech-in-noise perception. Given the unexpected findings of a group difference in ID, ID was introduced as a control variable in order to determine whether a significant group difference on RT was due to general cognitive demands related to task design or intensity-related processes rather than dynamic-related processes. This confirmed the group difference for RT discrimination, *F*_(1, 87)_ = 9.492, *p* = 0.012, partial η^2^ = 0.098, while FM remained insignificant, *F*_(1, 87)_ = 0.643, *p* = 1 (*p*-values are Bonferroni adjusted for multiple comparisons).

### Relations between literacy, phonological, and auditory skills

To assess the relations between subjects' literacy skills, phonological abilities and auditory processing skills, Pearson's correlation coefficients were calculated between the subjects' scores on measures of literacy, phonology, slow-rate dynamic auditory processing and speech-in-noise perception (lower left portion of Table 4). Phonological awareness was related to all measures of literacy, verbal short term memory and RAN, as well as RT and ID. Although FM was only found to relate to RT and ID, RT significantly correlated with measures of reading, spelling and measures of PA (spoonerisms and both RAN tasks).

**Table 4:** **Correlations among measures for auditory processing, speech perception, phonology and literacy skills, with (upper part) and without (lower part) controlling for group**.

**Measure**	**1**	**2**	**3**	**4**	**5**	**6**	**7**	**8**	**9**	**10**	**11**	**12**
1. Spell	–	0.366^***^	0.093	0.316^**^	0.385^***^	0.137	0.207(^*^)	0.065	0.061	−0.030	−0.123	0.033
2. Read	0.675^***^	–	0.227^*^	0.239^*^	0.323^**^	0.055	0.001	−0.073	0.146	0.064	−0.041	−0.010
3. DS	0.329^***^	0.404^***^	–	0.511^***^	0.301^**^	0.138	0.191	0.166	0.150	−0.128	0.117	0.147
4. NWR	0.521^**^	0.466^***^	0.591^***^	–	0.413^***^	0.170	0.194	0.071	−0.002	−0.042	0.106	0.275^*^
5. PA	0.700^***^	0.642^***^	0.457^**^	0.582^***^	–	298^**^	0.388^***^	−0.075	0.031	−0.221^*^	−0.028	0.224^*^
6. RANob	0.431^***^	0.356^**^	0.288^**^	0.349^**^	0.518^***^	–	0.722^***^	−0.255^*^	−0.014	−0.206	0.018	−0.042
7. RANcol	0.430^***^	0.280^*^	0.314^**^	0.346^**^	0.542^***^	0.775^***^	–	−0.175	0.081	−0.281^**^	−0.033	121
8. RT	−0.220^*^	−0.304^**^	0.005	−0.115	−0.314^**^	−0.382^***^	−0.301^**^	–	0.124	0.135	−0.108	−0.183
9. FM	−0.093	−0.042	0.057	−0.101	−0.132	−0.109	−0.015	0.211^*^	–	0.350^**^	−0.023	−0.033
10. ID	−0.241^***^	−0.173	−0.229^*^	−0.182	−0.375^***^	−0.321^***^	−0.249^*^	0.249^*^	0.402^***^	–	−0.047	−0.196
11. HINT	−0.112	−0.057	0.094	0.076	−0.047	−0.003	−0.046	−0.085	−0.015	−0.032	–	0.219^*^
12. CASPA	−0.024	−0.035	0.119	0.225^*^	0.132	−0.059	0.090	−0.165	−0.043	−0.181	0.220^*^	–

*Read, WRAT reading; Spell, WRAT spelling; DS, Digit Span; NWR, non-word recall; PA, Spoonerism; RANob, RAN object naming; RANcol, RAN color naming*.

* p < 0.05;

** p < 0.01;

*** p < 0.001; ^(*)^ Approaching significance of 0.05.

Since the correlational analyses showed that reading and spelling correlate with both PA and RT, the independent contribution of each was assessed through a multiple regression analyses with both RT and PA for predicting reading and spelling (see Table 5). Analyses showed that RT offers no unique influence to both literacy measure above that offered through PA.

**Table 5:** **Stepwise regressions showing the unique variance in the word reading, and spelling accounted for by PA and RT (*R*^2^**change and standardized Beta**)**.

**Step**	**Word reading**		**Spelling**	
	***R*^**2**^ change**	**β**	***R*^**2**^ change**	**β**
1. PA	0.412^***^	0.935	0.490^***^	0.983
2. RT	0.012	−0.171	0.000	−0.030

*** *p < 0.001*.

The addition of ID in the model to control for attention mechanisms produced the same pattern of results for reading, *F*_(3, 85)_ = 21.512, *p* < 0.001, *R*^2^ = 0.432, and spelling, *F*_(3, 85)_ = 27.258, *p* < 0.001, *R*^2^ = 0.490, as well as the addition of age and IQ with ID, *F*_(5, 83)_ = 13.802, *p* < 0.001, *R*^2^ = 0.454, and *F*_(5, 83)_ = 17.591, *p* < 0.001, *R*^2^ = 0.514.

Yet further investigation of RT's relationship with literacy within the dyslexic and the normal reading population did not reveal the same relationships present above. More specifically, the addition of group as a control measure to the regression model produced a larger significant contribution of PA, and none of RT, to reading, *F*_(6, 82)_ = 16.683, *p* < 0.001, *R*^2^ = 0.550 and spelling, *F*_(6, 82)_ = 23.392, *p* < 0.001, *R*^2^ = 0.631. In a similar vein, the other significant relationships that RT had across the entire population (lower left portion of Table 4) disappeared when controlling for group, with the exception of RAN object (upper right portion of Table 4).

### Individual deviance analyses

#### Individual differences

The examination of performance at the individual level in both the NR and DYS group allows for a better understanding of the proportion of individuals within each group showing poor performance on each measured variable, even when group differences are not found. Such analyses will also allow determining if any individual subject had consistent deviant performance across all levels of processing, or whether deviant performance is a more random occurrence indicating the involvement of influences different from an auditory perceptual deficit (Heath et al., 2006).

Individual performance of the *z*-scores of RT, FM, ID, CASPA, HINT, PA, RAN, and VSTM were analyzed. A deviancy threshold of −1.65 was used. Thus, any *z*-score falling below this threshold would be considered as deviant performance as described by Ramus et al. (2003) and subsequently used by Boets et al. (2006, 2007), Reid et al. (2007), and Hazan et al. (2009).

The number and proportion of deviant subject per group on each of the variables are presented in Table 6. All measures, with the exception of CASPA, HINT, ID, and FM, demonstrated a significantly higher portion of deviant subjects in the DYS group when compared with the NR group.

**Table 6:** **Individual deviancy analysis for each variable**.

**Measure**	**DYS**		**NR**		**χ^**2**^**	***p***
	***n***	**%**	***n***	**%**		
Literacy	31	86	0	0	70.932	<0.001
PA	26	72	1	2	50.184	<0.001
RAN	11	31	3	6	10.277	0.001
VSTM	19	53	2	4	29.079	<0.001
RT	21	58	12	22	12.129	<0.001
FM	11	31	8	15	3.213	0.073
ID	9	25	8	15	1.463	0.227
HINT	1	3	1	2	0.085	0.643
CASPA	5	14	9	17	0.127	0.722

*Where cells have expected count less than 5, the Fisher's Exact test p-values are reported*.

**Figure 1:**
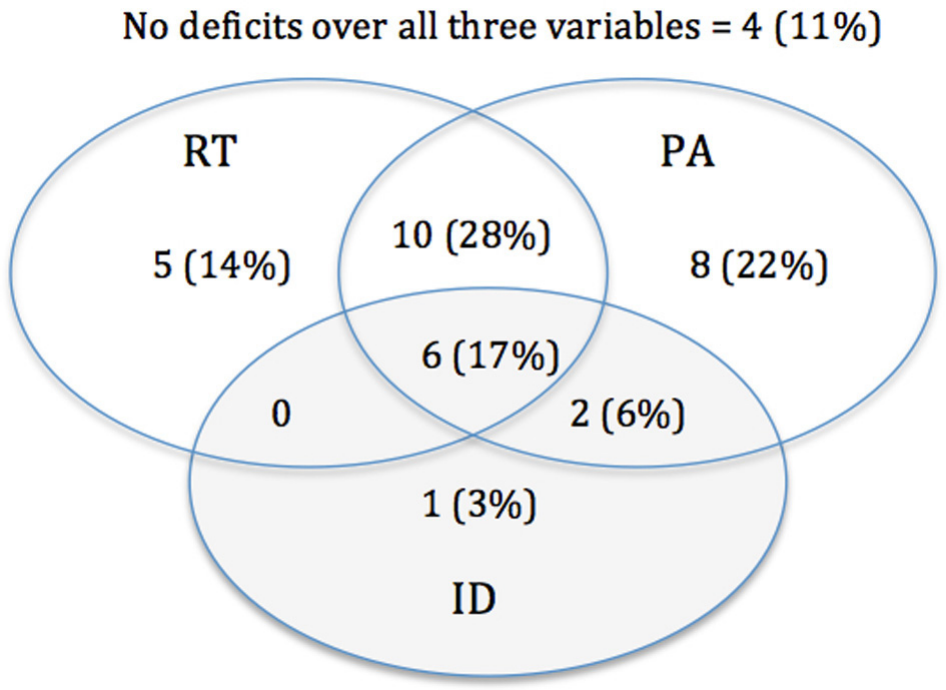
**Distribution of RT, PA, and ID deficits in the total sample of 36 dyslexic adult participants**. Measured in absolute numbers and percentages of impaired subjects.

An evaluation of subjects with at least one score deviating more than 1.65 *SD* for the various auditory, speech and phonological measures, demonstrated that deficits appeared inconsistent, with some subjects deviating only on one task, while others on two or three tasks. Due to the observation of a high percentage of deviancy found on measures of RT (58%) and PA (72%) within the dyslexic group, an exploration of the interrelation between deficiencies in these different skills were made. ID was included to address any questions of influence of task related demands and/or attention. Figure 1 shows the calculated number of subjects showing isolated vs. overlapping deficits. Results show that 28% of the dyslexic subjects possess a deficit in only PA (30% when controlled for ID), while 14% dyslexic subjects were found to only have a RT deficit (19% when controlled for ID). Dyslexic adults possessing an overlap in deficits were found to represent nearly half of the dyslexic subjects, 44% (37% when controlled for ID). Although a large percentage of overlap is present, the proportion of shared PA and RT deficit does not exceed the expected proportions represented within the whole dyslexic group. Investigation of the normal reading individuals revealed no overlap between deviancy of RT and PA, yet this might be due to a low number of deviant subjects.

## Discussion

It has been well established in the literature that dyslexic readers struggle with a phonological processing deficit and that such skills are related to literacy development and achievement (Snowling, 2000). Yet debate surrounds the question of whether this phonological processing impairment stems from a more primary deficit, such as a deficit in processing of speech sounds or due to a reduced sensitivity to slow-rate dynamic auditory information. This current study was set out to investigate speech perception and slow-rate dynamic auditory processing, in the form of RT and FM detection, in relation to phonological processing and literacy measures in dyslexic and normal reading adults.

### Slow-rate auditory processing deficit

In line with the auditory temporal processing deficit theory of dyslexia, we had expected our auditory measures of RT and FM to differentiate between dyslexic and non-dyslexic students but not our non-temporal auditory ID task.

With regard to the slow-rate auditory processing tasks, group analyses revealed significant differences between adults with dyslexia and normal readers in RT while the uncorrected *p*-value was found to be approaching significance in FM. The lack of a significant group difference for the FM measure was unexpected, since the majority of studies in dyslexic adults have demonstrated clear group differences (Witton et al., 1998, 2002; Ramus et al., 2003; Heath et al., 2006). With regard to RT, our results are in line with the bulk of previous studies demonstrating a lower performance in dyslexic children (e.g., Goswami et al., 2002; Fraser et al., 2010; Poelmans et al., 2011) and adults (e.g., Hämäläinen et al., 2005; Thomson et al., 2006; Pasquini et al., 2007), suggesting a RT-deficit across development and languages.

Plausible hypotheses to explain the unexpected finding of not finding a group difference for FM in the presence of a RT-deficit may be (1) low sensitivity of the behavioral measures used, (2) the influence of task demands and attention difficulties, or (3) specific characteristics of the auditory stimuli being used.

Stoodley et al. (2006) suggested that in a population, such as the one included in this study, psychophysical measures may not be sensitive enough to detect subtle auditory processing impairments due to possible compensation. They found dyslexic adults to be unimpaired in psychophysical FM discrimination tasks, yet group differences were found when electrophysiological recordings were used. In doing so, Stoodley and colleagues demonstrated that the inability to detect low level auditory processing deficits in some groups of high functioning dyslexics can be attributed to the task sensitivity and the level of compensation achieved by the individual. The lack of group differences for FM discrimination for our adult population differed from behavioral studies in pre-schoolers (Boets et al., 2007) and children (Poelmans et al., 2011), which employed similar methodologies and stimuli. Yet findings on the RT measures were found to be significant, which would not have been expected if Stoodley's theory of compensation influences is consistent across all psychophysical tasks, unless RT tasks offer greater sensitivity.

Criticism regarding the influence of task demand and complexity of psychophysical tasks (see Roach et al., 2004) could explain the inconsistency of these results and the unexpected group differences on the ID task. Of the 16 studies reviewed by Hämäläinen et al. (2013) that included a measure of ID, only two found a significant group difference between individuals with dyslexia and normal readers. In the only adult study which found a group difference in ID (Thomson et al., 2006), the authors attributed their findings to the task difficulty of their ID measure. Such findings of unexpected differences may support Roach et al.'s (2004) claim that poor performance and findings of group differences on psychophysical tasks are likely to be a function of attention and general task performance. In order to control for such task demand differences, ID was included in the statistical analyses as a control measure for all levels of analyses. After controlling for ID, group differences on RT remained present, indicating that this difference is rooted in processing stimuli-related properties differently rather than in attention differences.

Since our results do not clearly support the two explanations above, it is more likely that the pattern of results can be explained by a very specific deficit in slow-rate dynamic auditory processing. FM and RT tasks differ in how the auditory information is represented in the speech signal. As discussed by Rosen (1992), FM represents the fine structure of the speech waveform, while RT represents amplitude aspects of the speech envelope. The distinct pattern of results between RT and FM suggests that in adult dyslexics, the primary auditory dysfunction is more likely to be found in the perception of slow-rate dynamic auditory cues related to the speech envelope, as measured by RT, and not in the fine-structure, as measured by FM. Such findings reinforce previous studies in both child and adult populations (Goswami et al., 2002; Thomson et al., 2006; Fraser et al., 2010; Poelmans et al., 2011).

In sum, our results do not support a general deficit in slow-rate auditory processing of adult with dyslexia, yet, a subgroup of the adult dyslexic population may possess a more specific slow-rate dynamic processing deficit specific to the envelopes of the speech waveform.

### Speech-in-noise perception deficit in individuals with dyslexia

Slow-rate dynamic auditory cues are found in abundance in speech. It is believed that a deficit in the processing of these auditory cues, such as RT and FM, would ultimately lead to a disruption in speech perception.

Unlike the results of auditory processing, this present study was not able to demonstrate any evidence to support the continuation of the speech-processing deficit observed in youth (Snowling et al., 1986; Wible et al., 2002; Bradlow et al., 2003; Boets et al., 2007; Ziegler et al., 2005, 2009) into adulthood, suggesting developmental or task related influences. Although our speech masking stimuli were in line with previous studies with children, it may not have offered sufficient difficulty for use in an adult or a highly compensated population (Pennington et al., 1990). According to a recently published study by Dole et al. (2012), a stationary speech weighted background noise, as used in the present study, is less effective in differentiating between dyslexic and normal reading adults than modulated noises and background speech masks. Under the masking conditions of background speech or modulated noise an individual must rely on temporal dips in the masking noise to extract signals of the target speech signal (Howard-Jones and Rosen, 1993). It is thought that individuals with dyslexia may have difficulty perceiving these temporal dips, which is in line with our results of a RT deficit. Future studies should take into account Dole's findings to further assess the potential cascade of the RT difficulties observed in some dyslexics.

### Slow-rate auditory processing and speech perception relationship

Our findings showed significantly poorer performance in adult dyslexic readers on the RT task assessing slow-rate dynamic auditory processing, which relates to amplitude aspects of the speech envelope. If an indirect path of an RT deficit through speech perception existed, we would have expected to find a correlation with the sentence in noise measure that required a greater reliance on larger grain segmentation of the sentence stimuli. However, examination of the relationships between these variables could not clearly support this hypothesis. Yet, once controlled for group, CASPA was found to relate to phonological skills.

As discussed earlier, the use of stationary noise in our speech perception tasks may have limited our ability to find relationships with RT, which might be more closely related to speech perception in modulated noise. An alternative interpretation is that slow-rate auditory processing independently relates to reading related measures and not via speech perception measures. However, such a situation remains unlikely considering the prevalence of slow-rate dynamic auditory cues in the speech signal. Therefore one would expect to find a relationship between these two variables. Finally, Poelmans et al. (2011) offered an alternative explanation, stating that the lack of relationship could be a consequence of the fact that the developmental link between these variables diminishes over time and is no longer evident in later years.

Due to the lack of evidence found to support the relationship of auditory deficits and speech perception in adults, our results do not support the theoretical cascade effect of the auditory deficit through speech perception to one's phonological representations.

### Slow-rate dynamic auditory processing, phonological processing, and literacy

No significant correlations were found with FM nor with speech perception tasks. On the other hand, RT was found to correlate with measures of reading, spelling, phonological awareness and RAN, similar to findings of Thomson et al. (2006). Taking the regression analyses into account, it appears that any relationship between RT and reading is mediated through phonological processing and not speech-in-noise. These findings were similar to that of Pasquini et al. (2007). As discussed by Hämäläinen et al. (2005) it is highly improbable that the lower level skills of RT discrimination could be influenced by an individual's poor phonological awareness. Therefore, it is reasonable to assume that either this relationship reflects the same underlying perceptual deficit, or the ability to detect rapid changes in the speech envelope has a causal role in the development of PA. Although once controlled for group these relationships could no longer be supported, indicating that RT is not a good predictor of reading abilities in dyslexic or in normal readers. Yet, it is worth noting that a different pattern of findings might have emerged if a more direct assessment of decoding was employed, such as non-word reading measure (Hämäläinen et al., 2005).

Although the correlational analyses across all participants suggest interrelations between PA and RT, this finding should be nuanced at the individual level. When the prevalence and overlap of deviant performance on PA and RT was evaluated at the individual level, nearly half (45%) of the dyslexic population was found to possess a deficit in both, while 28 and 14% of the dyslexic population was found to have an isolated deficit in PA or RT, respectively (30 and 19% when controlled for ID). Despite co-occurrence in a large subsample of dyslexics, independence is suggested because the overlap between these variables is in proportion to what would be expected based on the frequency of each deficit in the total dyslexic group (i.e., 72% for a PA-deficit and 53% for a RT-deficit). Complemented with the lack of relationships once group was controlled for, it appears that phonological deficits seem not to be necessarily secondary to auditory problems since both deficits do not co-occur in every dyslexic subject. To increase our understanding, a longitudinal pre-reading study will be needed to assess the prevalence of the double deficit in RT and PA at earlier stages of reading development. In addition, training studies could help in verifying how one skill influences the other.

Given that in our adult study a large proportion of reading (problems) still remains unexplained, a multifactorial approach should be explored to fully identify the mechanisms underlying dyslexia. By investigating alternative cognitive factors, such as orthographic or morphological processing (Bekebrede et al., 2009), perceptual factors (Stein, 2001) and biological explanations (Nicolson et al., 2001), the variance and comorbid symptoms associated with the dyslexic population can be better understood.

### Limitations and implications

A limitation of this study was the sole inclusion of university students with dyslexia. It is reasonable to assume that by mere virtue of the fact that these young adults have reached university level education, varying levels of compensation are present in this specific group. Research has shown that the presence of relatively stronger cognitive abilities in some children with dyslexia allows for the minimization of parts of their phonological deficit later in life, allowing for the attainment of normal reading ability (Shaywitz et al., 2003). For example, a reliance or a strength in the use of contextual cues (Frith and Snowling, 1983; Nation and Snowling, 1998), semantic knowledge (Snowling et al., 2000), visual memory (Campbell and Butterworth, 1985), and morphological knowledge (Elbro and Arnbak, 1996) had been shown to aid in a dyslexic's ability to minimize the impact of the deficit in the expressed reading abilities. Stoodley et al. (2006) had also noted similar top down compensation processes influencing results of slow-rate dynamic auditory processing tasks (for a description of possible top down compensation processes see Pichora-Fuller, 2008). Therefore, percentages of observed deviant performance on slow-rate dynamic auditory processing tasks and phonological awareness measures could be underrepresented within our sample. Such potential levels of compensation limit our ability to extrapolate any findings to the general adult dyslexic population and could have potentially limited our ability in establishing clear group differences or correlations between variables. Having said this, our results do have implications in typifying the characteristics of dyslexic adults in higher education and broadening our understanding of how compensation may be expressed. This is especially relevant since accommodations are offered based on valid diagnosis given to them. Although the RT task sensitivity is lower than the phonological tasks' sensitivity, our result did demonstrate its potential to be included as an additional screening measure, for it was able to characterize a proportion of dyslexic adults not identified by a PA measure alone. Our data showed that purely relying on a PA tasks will result in missing a small subsample of dyslexics (in our study 14%).

A second implication is that a control task should be included. Our findings show the possible overestimation of the number of dyslexics when attention and task related demands are not accounted for. To avoid overestimation, future research should apply such a control task as presented in this paper, when designing a psychophysical testing battery and screening tools. Therefore, future development and study of this measure is still needed.

## Conclusion

In summary, our results suggest that the lower sensitivity to RT cues that was observed in dyslexic children is still observable in adulthood, while FM deficits are not. Hence, our results suggest that a general slow-rate dynamic auditory processing deficit may not be present within an adult dyslexic population, but may be confined to speech envelope cues rather than to fine structure. RT's influence on literacy outcomes was not direct and was found to be mediated through phonological processing (this relationship was lost once controlled for group). Unlike studies in younger children (Boets et al., 2006), the existence of speech-in-noise perception deficits and its mediating role in auditory processing and reading-related measures was not observed. Further research is needed in this area with attention to the selection of speech-in-noise masking stimuli and the sampling of a more diverse adult population, which does not primarily contain a university sample.

Although findings of a deficit in RT and its correlation with phonological skills are significant when examined across the entire population, many dyslexic subjects with a severe deficit in one of these skills were often found unimpaired in the other skills. At best, conclusions regarding the primary deficit of dyslexia being a slow-rate dynamic auditory processing deficit should be restricted to the processing of RT cues and can only be generalized to a subgroup of adults with dyslexia. Such a lack of consistency could implicate the necessity of a multifactorial model of dyslexia.

### Conflict of interest statement

The authors declare that the research was conducted in the absence of any commercial or financial relationships that could be construed as a potential conflict of interest.
